# Plant-Based Meat Alternatives Predicted by Theory of Planned Behavior Among Midwest Undergraduates

**DOI:** 10.3390/foods13233801

**Published:** 2024-11-26

**Authors:** Rachel H. Luong, Donna M. Winham, Mack C. Shelley, Abigail A. Glick

**Affiliations:** 1 Athletic Department, University of Minnesota, Minneapolis, MN 55455, USA; luong080@umn.edu; 2 Department of Food Science & Human Nutrition, Iowa State University, Ames, IA 50011, USA; aaglick@iastate.edu; 3 Departments of Political Science and Statistics, Iowa State University, Ames, IA 50011, USA; mshelley@iastate.edu

**Keywords:** plantbased meat alternatives, meat attitudes, young adults, subjective norms, consumer food behaviors, college students

## Abstract

Plant-based meat alternatives (PBMAs) such as the Impossible Burger^®^ imitate animal meat appearance, taste, feel, and texture. Part of their consumer appeal are the views that PBMAs are more environmentally friendly, reduce inhumane treatment of animals, and/or have preferred nutritional attributes. College-educated adults are one of the larger markets for these products. This cross-sectional online survey utilized the Theory of Planned Behavior to predict self-reported intakes of PBMAs among 536 undergraduates aged 18–25 at a Midwest university. Sixty-one percent had eaten PBMAs, and 17% wanted to try them. Twenty-two percent were uninterested non-consumers. Their top reason for not eating PBMAs was that they had no reason to decrease their meat intake. Multinomial logistic regression analysis showed subjective norms and positive attitudes about PBMAs increased the odds of more frequent intake, whereas non-consumers had less support from social contacts, but greater perceived behavioral control over general food access. Thus, those with supportive social influences, concerns about the environment, and animal welfare are more likely to consume PBMAs. More frequent PBMA consumption was observed among U.S.-born multicultural students, food insecure students, and those with less perceived behavioral control over food access. Future research should investigate the nuances between these associations further by examining the types of PBMAs consumed, their costs, and retail sources across student demographics.

## 1. Introduction

Plant-based meat alternatives (PBMAs) are designed to mimic animal meat’s appearance, taste, texture, and feel [1]. Consumers have stated PBMAs are chosen because of concerns for the environment, animal welfare, and the perception that they are healthier than meat [2,3,4]. A 2021 United States (U.S.) national survey showed that 65% of adults aged 18–80 had tried PBMAs [4]. Young adults with college degrees were more likely to have tried PBMAs than other age cohorts [4]. Sales for PBMAs were estimated at USD 1.2 billion with all plant-based foods totaling USD 8.1 billion for 2023. The market for these alternative products is expected to continue to grow, particularly among younger generations [5].

From an environmental standpoint, livestock production contributes to land degradation, water exhaustion, reduction of biodiversity, and increased greenhouse gases which accelerate climate change [6]. Xu et al. estimated that of 17.3 billion metric tons of carbon dioxide equivalents, animal-based food production was responsible for 57%, plant-based foods for 29%, and 14% for other utilizations [7]. While the public is increasingly aware of these environmental issues, such concerns may not be sufficient to prompt consumer behavior change at the point of purchase [8].

Animal well-being and welfare are important questions regarding meat consumption. Concentrated animal feeding operations (CAFOs) have come under increasing criticism by animal rights activists. Lack of disease prevention and veterinary treatment, management, appropriate shelter, nutrition, correct physical and mental needs, humane handling, and humane slaughter have all been noted in farmed livestock [9]. In the U.S. Midwest, animal meat production has been an important livelihood for generations of farmers. The state of Iowa is the top U.S. producer of pigs and eggs and ranks 10th in beef production [10,11]. Over 100 million animals are held in Iowa CAFOs [11]. Animal welfare and CAFO management are directly linked to environmental pollution from fecal material run-off [10]. Since animal husbandry and farming are strong cultural elements in much of Iowa, attitudes toward environmental degradation, animal welfare, and decreasing meat consumption may be ambivalent at best. Davitt et al. found that students from the College of Agriculture and Life Sciences at Iowa State University had more negative attitudes toward consuming PBMAs than those from other disciplines [12]. Speciesism or moral anthropocentrism may override people’s concern for animal welfare, especially when this attitude structure conflicts with economic concerns [8], the meal expectations of family members [13], or religious beliefs [14].

Meat continues to take center stage in American meals despite decades of Dietary Guidelines (DGA) recommendations to reduce intake to lower chronic disease risk [15]. The U.S. ranks within the top five countries with the highest annual meat consumption per capita [16]. In 2022, per capita meat consumption of beef, veal, pork, and sheep was 104.3 lbs. (47.3 kg) [16]. Argentina was the next highest country, with a per capita consumption of 95.2 lbs. (43.2 kg) per year. Other Western countries differed with the Canadian (75.5 lbs; 32.9 kg) and United Kingdom (62.2 lbs.; 28.2 kg) annual intake amounts substantially lower than the U.S. [16]. The DGA recommendation for red meat is no more than 12–18 ounces (0.75–1.1 lbs.) per week. Annual per capita data suggest Americans are consuming closer to 2 lbs. per week or double what is advised [15].

Consumption of processed or unprocessed red meat is associated with an increased risk of cardiovascular disease (CVD), total mortality, cancer, and type 2 diabetes [17,18,19,20]. Increasing red meat consumption by half a serving a day or more may increase mortality risk by 9–13% [17]. Type 2 diabetes risk was found to be increased by 15–27% in high meat consumption cases [18,19]. Thus, reducing meat consumption would lower the risks of at least four of the top 10 causes of death in the U.S. Heart disease remains the leading cause of death with cancer second. Stroke is the 5th cause. Type 2 diabetes is ranked 8th, but the disease contributes to comorbidities in CVD and renal diseases [18,20].

While the health risks of meat consumption drive some consumers to PBMAs, the debate about whether PBMAs are healthier than meat continues [1,2]. Meat is the major contributor of several nutrients such as zinc, protein, vitamin B6, vitamin B12, niacin, iron, and riboflavin [21]. PBMAs are often fortified with many of the vitamins and minerals found in meat to ensure dietary and nutritional needs are met or like those of meat products [22]. Most PBMA products are made to provide enough of these nutrients but some are short on others [22,23]. PBMAs may contain iron, zinc, and protein, but these nutrients typically are below the recommended daily value or found in a less bioavailable form, such as non-heme iron [22].

A comparison between the nutritional profile of the plant-based burger (Impossible Whopper™) vs. the animal-based beef burger (Whopper™) at the fast-food chain restaurant Burger King showed that the plant-based burger had fewer calories, fat, and cholesterol, and higher sodium, carbohydrates, fiber, and sugar, but the protein was nearly the same [24]. About 45% of participants in a national U.S.-based study believed PBMAs were healthier than animal meat, and 40% believed this to be true even after being given comparison nutrition labels and ingredients [1]. More research is needed to establish a conclusive statement about the nutritional quality of these products. Some researchers suggest that PBMAs be viewed as complementary to meat in terms of providing additional nutritional quality [23].

People with higher education levels were more likely to have tried PBMAs in a national survey [1]. In a 2019 Gallup poll, women and non-Whites reported consuming less meat in the previous year, while Midwesterners were less likely to have decreased meat consumption [25]. Higher household income was also associated with a greater likelihood of eating PBMAs [25]. A key demographic group driving climate, environmental, and social change in the U.S. is young adults between the ages of 18 and 25 [3,6]. 

A cross-sectional survey at Iowa State University in 2020 found that out-of-state students, those with a high fruit and vegetable intake, and vegetarians or vegans were significantly more likely to consume PBMAs than their peers [12]. As emerging adults in a new environment, college students experience rapid changes and adjustments in their social interactions, foodways, and roles with living on their own and away from the family unit [26,27,28]. Not all students are prepared to navigate social circumstances that may influence their food decisions [26]. Changing norms from the homes of students to those of their college peers and exposure to new ideas may influence attitudes and behaviors toward PBMAs [3,12,27,28].

Variables central to the theory of planned behavior (TPB) have demonstrated medium to large associations with dietary intentions and behaviors [29]. The TPB suggests that attitudes, subjective norms or motivations to comply, and perceived behavioral control interact synergistically to drive the intention to perform a behavior [30]. Attitudes can be in support of or against consuming a food or performing a given behavior. Subjective norms toward a behavior derive from approval or disapproval from social referents. Perceived behavioral control is influenced by belief or lack of belief in one’s ability to complete an action [31].

**Figure 1:**
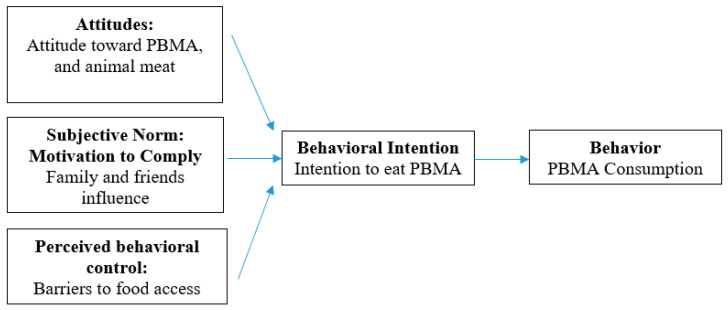
Theory of planned behavior conceptual framework for plant-based meat alternatives (PBMA) consumption.

From a broader standpoint, attitudes about meat consumption, animal welfare, environmental quality, climate change, nutrition, and health could influence attitudes toward and intakes of all foods, including PBMAs [2,3,12]. Subjective norms for food behaviors may shift from the parents and the family unit to peers, classmates, and instructors at college [27,28]. Accessibility of food retail outlets, transportation options, and cooking facilities are linked to perceived behavioral control for food preparation [28,32], and consequently the attainability of PBMA products. For college students, attitudes, subjective norms, and perceived behavioral control elements may change or evolve over the course of their education [26,27,33]. Figure 1 illustrates a conceptual framework of how the TPB constructs might influence PBMA consumption among university students.

The research objectives of this study were to: (1) assess the prevalence of PBMA consumption among undergraduate students; (2) describe associations between demographics, attitudes toward PBMAs, attitudes toward animal meat, and environmental concerns; and (3) develop a predictive model for PBMA consumption using the TPB. It was hypothesized that positive attitudes toward PBMAs, supportive subjective norms, and stronger perceived behavioral control would be more common among students who frequently eat PBMAs in contrast to those who do not. The current study expands upon the work of Davitt et al. by examining reasons for not choosing PBMAs, including a more ethnically diverse sample, and using TPB to frame the research [12].

## 2. Materials and Methods

### 2.1. Study Design and Sample Recruitment

The current study of PBMA consumption among undergraduates aged 18–25 was part of a larger project examining dietary acculturation, food intakes, and food patterns among U.S.-born White, U.S.-born multicultural (e.g., Hispanic, African American, Asian American), and international students aged 18–39 years. Results from the other research questions with a wider age range are reported elsewhere [34]. Prior to data collection, the survey was reviewed for face validity by two focus groups (10 food science majors; 8 students in a global nutrition course). Following suggested word changes from the focus groups, a different set of 11 students and 3 faculty members pilot tested the instrument. Minor changes were made in survey skip patterns and question wording before survey launch. The Registrar’s Office provided an email list for all students aged 18+ who had given permission to allow this information released (*n* = 28,211). Separate subgroup email lists for international students (*n* = 2387) and multicultural students (*n* = 4464) were provided. Survey invitations were sent to the international and multicultural students in early April 2022. Non-responders received a follow up email approximately one week later. In mid-April, a random sample of 65% of the complete student email list was generated using IBM SPSS software (Version 26, IBM, Armonk, NY, USA). No reminder emails were sent to the random sample. Email invitations, tracking, and data collection were conducted through Survey Monkey’s online platform (Momentive Inc., San Mateo, CA, USA). On average, the full survey took 14 min to finish. Responses were checked for completeness, consistency, and plausibility of answers. Participants who finished at least 75% of the survey, stated they wanted the incentive, and provided an email address received a USD 5 e-gift card to Amazon.com. 

### 2.2. Survey Development

Demographic characteristics included age, gender, state or country of origin, ethnic background, marital status, presence of children in the household, on-campus or off-campus housing, college, and whether or not the respondent had a campus meal plan [35]. Four items from a validated dietary fat screener were used to estimate consumption of ground meat, steaks or roasts, bacon or breakfast sausage, and ham or other cold cuts [36]. Frequency options were 1 time per month or less, 2–3 times per month, 1–2 times per week, 3–4 times per week, and 5 times or more per week. Food security status was assessed with the 10-item core food security module [37]. A dichotomous secure vs. insecure variable was computed following the USDA module instructions. Seven Likert statements that asked about the frequency of barriers to accessing foods in general, e.g., no time to shop (1 = very often; 5 = never) were used verbatim from the University of California Global Food Initiative survey [38]. The TPB construct of perceived behavioral control for overall food access was based these 7 items. A Likert statement on perception of diet change since starting at the university was drawn from the Stephenson Multigroup Acculturation Scale [39].

PBMA consumption variables were modeled after two U.S. national surveys by the International Food Information Council (IFIC) [1,4]. The following definition of PBMA was provided to respondents before they answered questions about them: “… foods like burgers, chicken, fish, sausages, and other products that attempt to mimic the flavor and texture of animal protein but are made with only plant products” [4]. Examples of companies and brands of these products were included as memory or recognition cues, e.g., ‘Impossible Burger’, to distinguish PBMAs from other plant proteins such as tofu or pulses. Seven PBMA consumption-level responses were provided (two or more times a day, once a day, weekly, monthly, at least once over the past year but not every month, not consumed over the past year and not interested in trying them, and not consumed in the past year but would like to try them) [4]. Those who had previously consumed or were interested in trying PBMAs were given a series of reasons why they did eat or theoretically would eat them. A similar series of response options were asked of those who did not want to consume PBMAs. Answer categories were used verbatim from the 2021 IFIC survey [4]. Participants were allowed to provide up to four responses for their behavior rationale plus a write-in comment option.

Attitudes toward PBMAs, meat, and environmental concerns were assessed with a series of 17 Likert scale statements (1 = strongly disagree; 5 = strongly agree). Four questions from the National Cancer Institute’s Food Attitudes and Behaviors Survey on subjective norms of self, family, and friends about ‘fruits and vegetables’ were adapted by substituting ‘plant-based meat alternatives’ in the phrasing [40]. Five questions about meat attitudes were used verbatim [40]. A question addressing the masculinity of eating PBMA was developed for the study based on social research views of food and gender roles [41,42]. Two statements on PBMAs as better for the environment, or better for health were adapted from IFIC [1]. Five attitude statements on animal welfare, bioengineered foods, sustainability of agriculture, religiosity, and nutritional quality of protein in PBMAs compared to meat were drawn from the 2021 IFIC survey [4].

### 2.3. Data Transformations and Analysis

Data were downloaded from Survey Monkey (Momentive Inc., San Mateo, CA, USA) in IBM SPSS Statistics format and subsequently analyzed using SPSS Version 26 (IBM, Armonk, NY, USA). All variables were examined for distribution normality. The seven options for frequency of PBMA consumption were condensed into four categories due to small sample sizes in three frequency options. The resulting four categories were defined as ‘frequent eaters’ who consumed PBMAs at least monthly, ‘infrequent eaters’ who have consumed PBMAs at least annually but not every month, the ‘interested in trying’ who have not yet consumed PBMAs, and the ‘non-eaters’ who do not want to try PBMAs. The five frequency options for the four animal meat consumption questions were summed to generate a continuous variable for regression analysis. The fifth category of ‘5 times or more per week’ was condensed with the ‘3–4 times per week’ option only for table display. 

The Chi-square test was used to examine for differences in demographic characteristics and food intake by respondents in the four levels of PBMA consumption. Analysis of Variance (ANOVA) with the post hoc Tukey multiple-comparison procedure was used to test for PBMA intake group differences for continuous variables including attitude statements and composite scales [43]. After principal components analysis, 13 of the 17 attitude statements were clustered into two attitude scales and one subjective norm scale following subsequent scale reliability testing. Four of the five questions for attitudes toward PBMAs were reverse-coded such that a higher value corresponded to a more positive attitude. The TPB construct of perceived behavior control was supported by a separate principal component analysis with the seven general food access barrier questions (not specific to PBMAs). The four TPB scales had Cronbach standardized alpha values ranging from 0.701 to 0.828 indicating good reliability [44]. Five attitude questions were retained as individual items in analysis (views toward animal welfare, bioengineered foods, diet change since at the university, eating of the same foods, and religiosity). The Cronbach’s alpha reliability values and resulting TPB scales are shown in Table 1.

A multinomial logistic regression (MLR) model estimated the influences of demographic and TPB constructs on reported PBMA consumption [43]. The reference category was the non-PBMA consumers. Screening for multicollinearity between variables using the Variance Inflation Factor did not detect any serious redundancy among the predictor variables. Gender, race/ethnicity, and food security status were entered as binary variables in the model. PBMA attitudes, attitudes toward animal meat, subjective norms toward PBMAs, five additional Likert statements, consumption frequency of animal meat, and perceived behavioral control scales were entered into the model as continuous variables.

## 3. Results

The survey response rates varied by the three types of email lists for the overarching project. The multicultural student response rate was ~8% (359/4417; 35 invalid emails, eight opt-outs, four duplicates). The international student response rate was higher, at 16.8% (392/2334; 17 invalid emails, nine opt-outs, one duplicate). For the random sample emails, the response rate was 2.4% (444/18,099; 163 invalid emails, 43 opt-outs, 32 duplicates). A net total of 1195 responses included 37 duplicates, 70 respondents who did not live near campus, 76 failed integrity checks, and 118 cases with incomplete PBMA variables. For the current PBMA analysis, individuals over age 25 (*n* = 243), and graduate students (*n* = 115) were excluded. Older undergraduates and graduate students have resources and living circumstances that are markedly different and would confound relationships among the relevant variables [28,32].

Pluralities of the 536 undergraduates were women (57.5%), lived off campus (57.6%), were not married (92.5%), and identified as U.S.-born White (48.1%). The sample had a mean age of 20.2 years. The overall ISU campus demographics were similar for Spring 2022 (female 55%, 51% living off campus, 76.9% White). Responses were highest from the College of Engineering (28.7%), Liberal Arts and Sciences (26.5%), and Agriculture (17.4%), with fewer responses from the smaller colleges of Business (13.1%), Human Sciences (8.8%), and Design (5.6%).

Table 2 shows demographic and meat intake characteristics by the four PBMA consumption categories. Women (*p* = 0.026), U.S.-born multicultural students, and vegetarians (both *p* < 0.001) had higher PBMA consumption frequencies. U.S.-born White students were a significantly higher percentage of the non-consumers (*p* < 0.001). Although a small portion overall (7%), almost 23% of those who reported being vegetarian or vegan were frequent PBMA consumers (*p* < 0.001). A higher percentage of frequent PBMA consumers were food insecure (*p* = 0.005) compared to the other groups. Although not significant, a higher percentage of Liberal Arts and Sciences students were infrequent or frequent PBMA consumers than respondents from other colleges. In contrast, Agriculture students were more often non-PBMA consumers. Non-consumers of PBMA had significantly more frequent intakes of ground meat, steak or roasts, bacon or sausage, or ham and cold cuts than the frequent PBMA consumers (*p* = 0.001; eta^2^ = 0.088). From 30 to 55% of the frequent PBMA consumers ate the four meat choices once per month or less. The mean meat consumption frequency score was 9.52 ± 3.2, ranging from 4 (once per month or less) to 19 (5+ times per week) for all respondents. The frequent PBMA consumers had lower meat intakes than their peers (*p* < 0.001).

The reasons given by students for choosing or wanting to choose PBMA are displayed in Table 3 by three frequency levels of intention or actual PBMA consumption. The top four reasons to eat or want to eat PBMA were liking to try new foods, heard a lot about them and were curious, thought it would taste good, and believe PBMA are better for the environment. The frequent eaters ranked taste highest with animal welfare, environmental concerns, and trying to eat less meat significantly higher than the other groups (*p* < 0.001). For the non-PBMA consumers, the majority said they were not trying to eat less meat (64.7%), did not think PBMA would taste good (52.1%), did not believe that PBMA were better for health than meat (44.5%), and viewed PBMA as too expensive (35.3%). Other reasons not to eat PBMA were that the ingredients seemed unappealing (20.1%), feeling that PBMA were too processed (14.3%), and not liking to try new foods (16.8%). Twenty percent said there was no specific reason.

Before developing the multinomial logistic regression (MLR) for PBMA consumption, the means of the four TPB scales and individual attitude items were compared. Table 4 shows the means of these variables with the referent category last to aid in the interpretation of the MLR. The four TPB scales were significantly different between PBMA consumption groups by one-way ANOVA (*p* < 0.001). The eta^2^ values ranged from 0.254 to 0.327 for the two attitude scales and the subjective norms scale. The non-PBMA consumers had the lowest scores (negative views) on the PBMA attitude and subjective norms scales (less social support for PBMAs), and the highest scores on the meat attitude scale (positive view). Non-PBMA consumers had the highest scores on the perceived behavioral control scale, indicating the least barriers to general food access (*p* < 0.001; eta^2^ = 0.050). ANOVA revealed significant differences by PBMA intakes for the five individual attitude statements as well. Agreement about the importance of animal welfare in food production (*p* = 0.015; eta^2^ = 0.019) and change in diet since coming to the university (*p* = 0.001; eta^2^ = 0.032) were both lowest among non-PBMA eaters (less concern; no diet change) and highest for frequent PBMA consumers (more concern; more diet change). The non-PBMA eaters were significantly more likely to agree they were religious (*p* < 0.001; eta^2^ = 0.035) than the other three groups. A significant portion of the frequent PBMA consumers disagreed that they ate the same foods (*p* < 0.001; eta^2^ = 0.042), and disagreed they did not want to eat bioengineered or genetically modified foods (*p* < 0.001; eta^2^ = 0.039). 

The most conservative MLR model showed significant reductions in the likelihood of not eating PBMA (the reference category) from the TPB constructs of attitudes toward PBMAs, and subjective norms about PBMAs. A 1-unit increase in positive PBMA attitudes was associated with a 14% decrease in the likelihood (odds ratio, or OR) of not eating PBMA (OR 0.857; 95% CI, 0.806 to 0.911), Wald χ2(1) = 24.375, *p* < 0.001. For the subjective norms scale, a 1-unit increase in positive subjective norms about PBMA was associated with a 23% decrease in the likelihood of not eating PBMA (OR 0.774; 95% CI 0.726 to 0.825; Wald χ2(1) = 61.894, *p* < 0.001). A 1-unit increase in positive attitudes toward meat increased the likelihood of not eating PBMA by about 10% (OR 1.105; 95% CI 1.042 to 1.172; Wald χ2(1) = 10.312, *p* < 0.001). Similarly, a 1-unit increase in perceived behavioral control was associated with about a 4% increase in the likelihood of not eating PBMA (OR 1.044; 95% CI 1.015 to 1.074; Wald χ2(1) = 8.768, *p* =0.003). Persons who ate the same foods all the time were 17% more likely not to eat PBMA (OR 1.170; 95% CI 1.005 to 1.361; Wald χ2(1) = 4.119, *p* < 0.042). Those who preferred not to eat bioengineered foods were also about 17% more likely not to consume PBMA (OR 1.172; 95% CI 1.005 to 1.367; Wald χ2(1) = 4.074, *p* < 0.044) (Table 5).

## 4. Discussion

This study assessed the relationship of consumption of PBMAs among undergraduates, with demographics, attitudes toward PBMAs, attitudes toward animal meat, and environmental concerns using the TPB. Based on these findings a MLR was estimated to predict PBMA consumption using the TPB. The findings support our initial hypothesis that improved attitudes, and subjective norms, with respect to PBMA improved the likelihood of undergraduate students consuming these foods. However, perceived behavioral control’s influence on PBMA was in the reverse direction than hypothesized. These data provide a more comprehensive picture of the subtleties between non-consumers, those who want to try PBMAs, infrequent PBMA consumers, and frequent PBMA consumers. 

The first objective was to describe PBMA intake patterns among this population. About 60% of student respondents had eaten PBMAs at least in the past year, which was similar to the 55% of students aged 18–30 reporting from the same campus in 2020 [12]. Data from U.S. national surveys indicate about 62% of adults aged 18–44 in 2020 [1], and 65% of adults aged 18–80 in 2021 had eaten PBMAs before [4]. Gender differences in consumption frequency were observed in this study, with more women than men reporting more frequent PBMAs. This observation follows similar national-level trends where women were more likely than men to buy and consume PBMAs [24]. Vegetarians and vegans, although only 7% of respondents, were about a quarter of the frequent PBMA consumers. Other studies have suggested that vegetarians are more likely to consume plant-based foods that do not resemble animal meat [45]. Two unexpected demographic trends emerged from these data. One was a higher per-centage of multicultural students who reported eating PBMA frequently. The second was a higher percentage of food insecure students in the frequent PBMA consumption group. The current study did not ask what types of PBMA were being consumed or their source (fast-food outlet, retail grocer, campus meals). Future research should provide more details on the types and sources of PBMAs eaten to add clarity to consumer practices and preferences.

The second study objective was to describe reasons given for trying or not trying PBMAs. The top three responses (liking to try new foods, hearing about them and being curious, thought would taste good) for trying PBMAs were identical to those observed in Davitt et al. [12]. The top two were the same as the 2020 national survey by IFIC [1]. The 2021 national IFIC survey on PBMA consumption did not use a comparable question. Trying to eat less meat was the 7th ranked response in the current study but was the 3rd most frequent reason for the 2020 IFIC cohort [1]. This finding suggests that college students who chose PBMA were not all excluding animal meat from their diets. Comparisons between the want to eat, infrequent eaters, and frequent eaters showed that eating less meat, views on PBMA as better for the environment and one’s health, and made without harming animals, were more important to those with higher intakes of PBMA. Davitt et al. found similar results in their analysis of PBMA intakes as a dichotomous variable. For those who want to try them or choose them infrequently, the novelty of the product and encouragement of others may be more persuasive than environmental or health advocacy alone [3,8]. 

In contrast, almost 65% of the non-PBMA consumers said they were not trying to eat less meat as the top reason why they were not interested in them. Over 50% of the non-consumers thought PBMA would not taste good. The 2020 IFIC non-PBMA sample also agreed they thought PBMA would not taste good (31%) and that they were not trying to eat less meat (19%) [1]. The 2020 Davitt study did not ask about reasons why PBMA was not chosen [12].

It is possible that those not interested in trying PBMA view them more as an implied replacement for animal meat whereas those who consume PBMA view them as a complementary food. One non-consumer wrote in an optional comment, “I don’t like how PBMA try to pass themselves off as a faux-meat. They should market for their target audience of vegans and vegetarians, not meat eaters.” Religiosity or speciesism may play a role in viewing animals as different [14]. Indeed, nuances were observed between PBMA and animal meat consumption levels among respondents. The frequent PBMA consumers reported eating animal meats less often, but 45–70% of them ate meat at least several times per month. While half of non-consumers disagreed that PBMAs were for vegetarians only, over 80% of the frequent PBMA consumers disagreed. Perhaps most telling was the observation that two-thirds of the non-consumers viewed PBMA as ‘weird,’ with only 11% of the frequent PBMA consumers sharing this attitude. The observation that multicultural, and food insecure students had frequent PBMA consumption more than other groups was unexpected. This phenomenon needs further investigation to determine if it is a sampling artifact. It is possible these foods were priced lower at the time of the survey, were readily available at campus and other food outlets, or these respondents were more interested in the qualities PBMA has to offer. 

Comparative responses for these questions from Davitt [12] or either IFIC survey [1,4] are not available. Previous research has indicated that people who prefer animal meat would like PBMA which looks, tastes, and feels like the real product [45,46]. Michel et al. [45] pointed out that the social setting, or subjective norms, have a strong effect on meat vs. meat alternative acceptability based on a German survey. College students are heavily influenced by their peers and program of study. Differences among college enrollment in these data suggest similar trends to Davitt et al., suggesting lower PBMA consumption in Agriculture or Veterinary-related majors [12]. The study setting was a land-grant university with a strong agricultural program, including livestock breeding. Further exploration of agrarian or pro-farmer views in relation to PBMAs might clarify these points.

As expected, the influence of TPB variables showed that frequent PBMA consumers viewed PBMAs more positively than less frequent, want to try, and non-consumer respondents. Attitudes toward meat followed the expected trend, but as mentioned above the frequent PBMA consumers were not all ‘anti-meat’ in their attitude statements either. However, there was more adamancy about the importance of meat by the non-consumers. Other studies have indicated similar results. Those with a higher meat attachment or positive bond towards meat consumption are less likely to change eating habits and adopt a more plant-based diet [47]. Those with an attitude in support of PBMAs are more likely to be consumers [12].

The third objective of the study was to investigate the influence of TPB constructs on PBMA consumption. Our findings support the hypothesis that positive attitudes towards PBMAs improved subjective norms from support systems, and enhanced perceived behavior control would positively influence the likelihood of consuming PBMA. Both subjective norms and positive attitudes toward PBMAs were the strongest predictors of PBMA consumption. Thus, it may be reasonable to suggest that those with cultural and environmental beliefs supporting PBMA consumption, as well as support systems that share similar consumption patterns and beliefs, may be the biggest determinants in one’s choice to consume these foods. Our findings indicate barriers to food access may influence this decision. Future research should examine whether availability, access, and barriers with respect to PBMA may affect consumption. As PBMA consumption increases nationwide, colleges and universities may benefit from incorporating these options for students who prefer them.

There are several limitations to the study. Data on the specific consumption or purchasing behavior of the types of PBMA and sources were not collected. The perceived behavioral control or food access questions were general and not specific to locating PBMA items. Despite wording to clarify the definition of PBMA as products that mimic animal meat, some respondents may have included other plant-based foods in general in their responses. Although differences in chronological age were not significant, we were unable to analyze data by academic year. It is possible that influences on freshmen are different than for seniors. The findings are not generalizable beyond the study sample.

## 5. Conclusions

This study provides further information on the factors that influence college undergraduate’s decisions to consume PBMAs. These results indicate that those with supportive social influences, concerns about the environment, and animal welfare are more likely to consume PBMAs. More frequent PBMA consumption was observed among U.S.-born multicultural students, food insecure students, and those with less perceived behavioral control over food access. Studies have shown that young adults have greater climate and social concerns compared to other groups, making these individuals candidates for PBMA consumption. Our results support the perception that environmental concern may influence PBMA consumption, in addition to support from social circles. While barriers to food acquisition in general play a role, future research should investigate the nuances between these associations further by examining the types of PBMAs consumed, their costs, and retail sources across student demographics. 

## Figures and Tables

**Table 1:** Theory of Planned Behavior (TPB) scales and Likert statements used in regression model (*n* = 536).

Responses: 1—Strongly Disagree, 2—Disagree, 3—Neither Agree or Disagree, 4—Agree, 5—Strongly Agree	Mean ± SD
Attitude toward Plant-Based Meat Alternatives (PBMAs) (Range 5–25; higher score = favor PBMAs)	18.03 ± 3.62
PBMAs are not a masculine food (Reverse Coded = RC)	4.06 ± 1.06
Only vegetarians or vegans eat PBMAs (RC)	3.89 ± 1.03
PBMAs are better for the environment	3.45 ± 1.04
PBMA foods seem ‘weird’ (RC)	3.35 ± 1.21
Not important if foods are produced in an environmentally sustainable way (RC)	3.27 ± 1.00
Cronbach’s adjusted alpha = 0.701	
Attitude toward Animal Meat (Range 4–20; higher score = favor meat)	12.11 ± 3.81
I think meals should include meat	3.32 ± 1.14
Dinner does not seem right without meat	3.15 ± 1.32
Protein quality of PBMAs is not as good as real meat	3.04 ± 1.09
After I eat a meal without meat, I still feel hungry	2.60 ± 1.14
Cronbach’s adjusted alpha = 0.828	
Subjective Norms for PBMAs (Range 4–20; higher score = more support)	8.68 ± 3.33
My family or friends would be willing to eat PBMAs	2.93 ± 1.13
I often encourage my family and friends to eat PBMAs	2.02 ± 1.09
My family and friends encourage me to eat PBMAs	1.95 ± 1.01
My family and friends often eat PBMA when we are together	1.79 ± 1.01
Cronbach’s adjusted alpha = 0.791	
Individual Likert statements included in Ordinal Logistic Regression (Range 1–5)	
Animal welfare in food production is important	3.86 ± 0.89
My diet has changed since I came to the university	3.79 ± 1.05
When it comes to food, I eat the same things all the time	3.44 ± 1.13
I consider myself to be religious	2.82 ± 1.46
I prefer to not eat bioengineered or genetically modified foods	2.67 ± 1.15
Responses: 1—Very often, 2—Often, 3—Sometimes, 4—Rarely, 5—Never;	
Perceived Behavioral Control (Range 7–35; higher score = more control)	24.54 ± 6.14
No time to prepare food	2.85 ± 1.28
No time to shop for food	3.19 ± 1.31
Cost of food	3.36 ± 1.29
Hours of operation of campus food outlets	3.56 ± 1.30
Unavailability of cultural or ethnic food items	3.78 ± 1.44
Location of campus food outlets	3.80 ± 1.27
Lack of reliable transportation to food outlets	4.00 ± 1.24
Cronbach’s adjusted alpha = 0.798	

**Table 2:** Demographic and diet characteristics of 18–25-year-old Midwest university undergraduate students by consumption frequency of plant-based meat alternatives (PBMAs).

Demographics	Total (*n* = 536)	Not Eat (22.2%, 119)	Wants to Eat (17.2%, 92)	Infrequent Eater (33.8%, 181)	Frequent Eater (26.9%, 144)	*p*
	←————————— % —————————→					
Gender						0.026
Man	42.5	50.4 _a_	45.7 _a_	43.6 _a_	32.6 _b_	
Woman	57.5	49.6 _a_	54.3 _a_	56.4 _a_	67.4 _b_	
Ethnic-cultural origins						<0.001
White-US-born	49.1	67.2 _a_	53.3 _b_	42.5 _bc_	39.6 _c_	
Multicultural—US-born	36.8	24.4 _a_	38.0 _b_	38.7 _b_	43.8 _b_	
International	14.2	8.4 _a_	8.7 _ab_	18.8 _c_	16.7 _bc_	
Vegetarian/Vegan						<0.001
No	92.7	98.3 _a_	100 _a_	97.8 _a_	77.7 _b_	
Yes	7.3	1.7 _a_	0 _a_	2.2 _a_	22.9 _b_	
Food Security Status						0.005
Secure	70.0	79.7 _a_	71.7 _ab_	71.1 _a_	59.7 _b_	
Insecure	30.0	20.3 _a_	28.3 _ab_	28.9 _a_	40.3 _b_	
College						n.s.
Engineering	28.7	29.4 _a_	29.3 _a_	29.3 _a_	27.1 _a_	
Liberal Arts and Sciences	26.5	16.8 _a_	25.0 _ab_	28.7 _b_	32.6 _b_	
Agriculture	17.4	26.9 _a_	9.8 _b_	16.6 _b_	15.3 _b_	
Business	13.1	12.6 _a_	17.4 _a_	13.3 _a_	10.4 _a_	
Human Sciences	8.8	8.4 _a_	10.9 _a_	8.3 _a_	8.3 _a_	
Design	5.6	5.9 _a_	7.6 _a_	3.9 _a_	6.3 _a_	
Meat and meat product consumption frequencies						
Ground meat eaten						<0.001
Once per month or less	15.7	8.4 _a_	12.0 _a_	10.5 _a_	30.6 _b_	
2–3 times per month	23.1	20.2 _a_	27.2 _a_	24.9 _a_	20.8 _a_	
1–2 times per week	36.4	36.1 _a_	33.7 _a_	39.8 _a_	34.0 _a_	
3+ times per week	24.8	35.3 _a_	27.2 _a_	24.9 _a_	14.6 _b_	
Steak or roasts eaten						<0.001
Once per month or less	24.6	10.1 _a_	20.7 _b_	21.5 _b_	43.1 _c_	
2–3 times per month	30.0	27.7 _a_	39.1 _b_	30.4 _ab_	25.7 _a_	
1–2 times per week	25.7	28.6 _a_	21.7 _a_	28.7 _a_	22.2 _a_	
3+ times per week	19.6	33.6 _a_	18.5 _b_	19.3 _b_	9.0 _c_	
Bacon, sausage eaten						0.008
Once per month or less	41.8	30.3 _a_	42.4 _a_	38.1 _a_	55.6 _b_	
2–3 times per month	28.7	33.6 _a_	29.3 _ab_	31.5 _a_	20.8 _b_	
1–2 times per week	20.0	26.9 _a_	16.3 _a_	19.3 _a_	17.4 _a_	
3+ times per week	9.5	9.2 _a_	12.0 _a_	11.0 _a_	6.3 _a_	
Ham and cold cuts eaten						0.036
Once per month or less	31.2	24.4 _a_	27.2 _a_	28.2 _a_	43.1 _b_	
2–3 times per month	25.9	23.5 _a_	30.4 _a_	27.6 _a_	22.9 _a_	
1–2 times per week	25.0	28.6 _a_	23.9 _a_	24.9 _a_	22.9 _a_	
3+ times per week	17.9	23.5 _a_	18.5 _ab_	19.3 _a_	11.1 _b_	
Meat consumption score ^1^ (µ ± SD) (higher score = more intake)	9.52 ± 3.2	9.60 ± 3.2	9.52 ± 3.0	9.77 ± 2.8	8.12 ± 3.3	<0.001

Each subscript letter (a, b, etc.) denotes a subset of PBMA consumption categories whose column percentages do not differ significantly from each other at the 0.05 level; n.s. = not significant. ^1^ Meat consumption score is sum of the four meat category frequencies.

**Table 3:** Reasons for eating a plant-based meat alternative (PBMA) among Midwest university undergraduate students aged 18–25 years who had tried or wanted to try them.

Why Did You Decide to Eat a Plant-Based Meat Alternative? ^1^	Total (*n* = 417)	Wants to Eat (22.1%, 92)	Infrequent Eater (43.4%, 181)	Frequent Eater (34.5%, 144)	*p*
	←————————— % —————————→				
I like to try new foods	51.6	54.3 _ab_	57.5 _b_	42.4 _a_	0.021
Heard a lot about them and was curious	39.6	59.8 _a_	43.1 _b_	22.2 _c_	<0.001
Thought it would taste good	31.2	20.7 _a_	27.1 _a_	43.1 _b_	<0.001
Believe plant alternatives are better for the environment	30.2	28.3 _a_	21.5 _a_	42.4 _b_	<0.001
Made without harming animals	24.9	28.3 _a_	15.5 _b_	34.7 _a_	<0.001
Encouraged to try by friends or family	22.8	10.9 _a_	29.8 _b_	21.5 _b_	0.002
Trying to eat less meat	22.5	19.6 _a_	12.7 _a_	36.8 _b_	<0.001
Believe plant alternatives are better for health	22.3	18.5 _a_	11.0 _a_	38.9 _b_	<0.001
Reasonably priced	16.5	14.1	17.1	17.4	n.s.
Ingredients intrigued me	16.3	21.7	15.5	13.9	n.s.
On a menu of a restaurant I like	11.0	5.4	13.3	11.8	n.s.
No specific reason	9.8	9.8	13.3	5.6	n.s.
Noticed in the meat aisle of the store	5.0	6.5	5.5	3.5	n.s.

^1^ Could select up to four responses. Thus, the numbers will not add up to 100% in each column. Each subscript letter (a, b, etc.) denotes a subset of PBMA consumption categories whose column percentages do not differ significantly from each other at the 0.05 level Questions modified from International Food Information Council (IFIC) Foundation. A Consumer Survey on Plant Alternatives to Animal Meat. 2020 [1,4]. n.s. = not significant.

**Table 4:** Means of Theory of Planned Behavior scales and attitude statements by PBMA consumption levels used in multinomial logistic regression (MLR) model among Midwest undergraduates ^1^.

	Total (536)	Frequent Eater (26.9%, 144)	Infrequent Eater (33.8%, 181)	Wants to Eat (17.2%, 92)	Not Eat (22.2%, 119)	*p*
*TPB Scales*	←————————— µ ± SD —————————→					
PBMA attitudes	18.03 ± 3.6	19.99 ± 2.9	18.52 ± 3.0	18.42 ± 3.3	14.61 ± 3.2	<0.001
Meat attitudes	12.11 ± 3.8	9.49 ± 3.6	12.23 ± 3.2	12.23 ± 3.1	14.99 ± 3.2	<0.001
Subjective norms	8.68 ± 3.3	11.4 ± 3.1	8.68 ± 2.9	8.18 ± 2.7	6.0 ± 2.1	<0.001
Perceived behavioral control	24.54 ± 6.1	22.58 ± 6.3	24.61 ± 5.9	24.98 ± 6.1	26.5 ± 5.8	<0.001
*Individual attitude statements*						
Animal welfare in food production is important	3.86 ± 0.9	4.00 ± 0.9	3.87 ± 0.8	3.88 ± 0.9	3.65 ± 1.0	0.015
My diet has changed at college	3.79 ± 1.0	3.94 ± 1.0	3.88 ± 0.9	3.84 ± 1.0	3.45 ± 1.2	0.001
Eat the same things all the time	3.44 ± 1.1	3.13 ± 1.1	3.40 ± 1.1	3.76 ± 1.1	3.64 ± 1.0	<0.001
Consider myself to be religious	2.82 ± 1.5	2.48 ± 1.4	2.88 ± 1.4	2.68 ± 1.5	3.24 ± 1.5	<0.001
Prefer to not eat bioengineered or genetically modified foods	2.67 ± 1.2	2.46 ± 1.1	2.52 ± 1.0	2.79 ± 1.3	3.03 ± 1.2	<0.001

^1^ Note that the referent category for MLR analysis is ‘does not eat PBMA’.

**Table 5:** Multinomial logistic regression model for predictors of plant-based meat alternatives (PBMAs) consumption by Midwest undergraduates (reference group is those who do not eat PBMAs).

			95% Confidence Interval for Odds Ratio		
	B (SE)	Sig.	Lower	Odds Ratio	Upper
PBMA Levels of Consumption					
Level 1 (Frequent eater)	−3.258 (1.0144)	0.001	0.005	0.038	0.281
Level 2 (Infrequent eater)	−1.202 (1.0071)	0.223	0.042	0.301	2.163
Level 3 (Wants to eat)	−0.026 (1.0051)	0.979	0.136	0.974	6.986
Attitudes					
PBMA attitude scale	−0.154 (0.0312)	<0.001	0.806	0.857	0.911
Animal meat attitude scale	0.100 (0.0301)	0.001	1.042	1.105	1.172
Subjective Norms					
PBMA subjective norm	−0.257 (0.0326)	<0.001	0.726	0.774	0.825
Behavioral Control					
Perceived behavioral control	0.043 (0.0144)	0.003	1.015	1.044	1.074
Other Beliefs/Attitudes					
Eat the same foods most times	0.157 (0.0773)	0.042	1.005	1.170	1.361
Not eat bioengineered food	0.158 (0.0785)	0.044	1.005	1.172	1.367

## Data Availability

The data are available upon request from the corresponding author.
